# Offspring long-term infectious morbidity following pregnancies with cervical cerclage

**DOI:** 10.1007/s00404-026-08431-1

**Published:** 2026-04-27

**Authors:** Amir Snir, Polina Schwarzman, Tamar Wainstock, Eyal Sheiner

**Affiliations:** 1 The Obstetrics and Gynecology Division, Soroka University Medical Center, Ben-Gurion University of the Negev, POB 151, Beer-Sheva, Israel; 2 The Department of Public Health, Faculty of Health Sciences, Ben-Gurion University of the Negev, Beer-Sheva, Israel

**Keywords:** Cervical cerclage, Long-term infectious morbidity, Maternal–fetal immunology

## Abstract

**Introduction:**

Cervical cerclage is an acceptable procedure in women with cervical insufficiency and is known to be effective in the prevention of preterm delivery. However, limited data exist regarding long-term health outcomes among offspring exposed to cerclage during pregnancy. Since the presence of a foreign body during pregnancy may change the vaginal microbiome, we aimed to study whether a cervical cerclage is associated with long-term infectious morbidity of the offspring.

**Study design:**

A retrospective population-based cohort study was performed at a tertiary medical center, including all singleton deliveries between the years 1991–2021. Long-term infectious morbidity was compared among offspring after pregnancies with and without cervical cerclage. The diagnoses of infectious morbidities were defined based on ICD-9 codes as recorded in community clinics and hospitalization files. A Kaplan–Meier survival curve was utilized to evaluate the cumulative incidence. A Cox proportional hazards model was used to control for confounders.

**Results:**

Out of 356,356 offspring included in the analysis, 0.4% (*n* = 1416) were following pregnancies with cervical cerclage. Unadjusted analyses demonstrated no significant difference in total infectious morbidity between the groups (OR 1.0, 95% CI 0.9–1.1; *p* = 0.369, Table 1). Kaplan–Meier analysis showed no difference in cumulative incidence (log-rank test *P*-value = 0.19, Fig. 1). In the primary analysis, cerclage was not associated with long-term infectious morbidity. However, in a secondary model, after adjustment for confounders including gestational age, obesity and diabetes, cerclage exposure was associated with a modest reduction in the risk of long-term infectious morbidity (adjusted HR 0.9, 95% CI 0.87–0.99, *p* = 0.036).

**Conclusion:**

In this large population-based cohort, cervical cerclage was not associated with increased long-term infectious morbidity in offspring. A modest association with reduced infectious morbidity was observed after adjustment for confounding factors. These findings should be interpreted cautiously given the observational design and potential residual confounding.

## What does this study add to the clinical work

**Table:** 

This large population-based study suggests that cervical cerclage is not associated with increased long-tern infectious morbidity in offspring and may even confer a modest protective effect. These findings support the clinical safety of cerclage regarding offspring infectious outcomes and may reassure clinicians when considering this intervention.	

## Introduction

Cervical cerclage is a vastly utilized intervention aimed at reducing the risk of preterm birth in women with a history of cervical insufficiency or by certain sonographic or physical exam findings [1]. Cervical cerclage reduces the risk of preterm birth by approximately 20% in these women [2, 3]. Cerclage placement in relevant cases is recommended by both the American and the UK Royal College of Obstetricians and Gynecologists [4, 5].

Despite its established effectiveness and benefits in reducing preterm births and improving neonatal outcomes, there is a paucity of evidence regarding potential long-term health implications as outcomes beyond the neonatal period are scarcely investigated. In the two Cochrane reviews [3, 6] that evaluated the effectiveness of cervical cerclage for preventing preterm birth, no studies were identified assessing long-term outcomes, emphasizing the importance that comprehensive research in this area be conducted. Cervical cerclage has been studied broadly for its traditional benefits in prolonging gestation and improving neonatal outcomes primarily in the context of mechanical support to the cervix [3]. However, the presence of a cervical suture and its potential interaction with the vaginal microbiome and cervical immune environment raise questions regarding possible biological effects beyond mechanical support.

Several biological mechanisms could theoretically link cerclage exposure to long-term health outcomes in offspring. The presence of a cervical suture introduces a foreign material that may trigger localized immune responses in cervical tissue. Such responses may alter the local inflammatory milieu or microbial environment during pregnancy, although the clinical implications of these changes remain uncertain. [7–9]

Conversely, cerclage may indirectly improve long-term outcomes through prolongation of gestation. Prematurity is a well-established risk factor for increased infectious morbidity during infancy and childhood, and even modest increases in gestational age may improve immune maturation and reduce infection risk [10–15]. Therefore, it is plausible that cerclage could influence long-term infectious morbidity through mechanisms related to gestational age rather than direct immunological effects.

Given the limited available evidence regarding long-term outcomes, we aimed to evaluate the association between cervical cerclage during pregnancy and long-term infectious morbidity in offspring using a large population-based cohort with extended follow-up.

## Materials and methods

This retrospective population-based cohort study included all singleton deliveries that occurred at Soroka University Medical Center (SUMC) between 1991 and 2021. SUMC is the only tertiary hospital in southern Israel and serves a population exceeding one million residents, providing a comprehensive regional database of obstetric and pediatric outcomes. Multiple gestations were excluded from the analysis. Offspring were categorized according to whether the pregnancy was complicated by cervical cerclage placement. Preterm birth was defined as delivery before 37 completed weeks of gestation. For descriptive purposes, preterm birth was further categorized into 28–34 weeks and 34–37 weeks of gestation. The study was approved by the institutional review board in accordance with the Helsinki Declaration. Due to the retrospective nature of the study, informed consent was waived by the institutional review board.

Infectious morbidities were compared among offspring with and without placement of cervical cerclage during the pregnancy. Follow-up was conducted up to the age of 18 years. Infectious morbidity cases were identified through diagnoses coded under the International Classification of Diseases, Ninth Revision (ICD-9), extracted from CHS outpatient clinic and hospital records. The infectious disease may have been the main reason for admission or a background disorder in the file. The infectious categories evaluated included respiratory infections, viral infections, ophthalmic infections, skin infections, bacterial infections, neonatal infections, bacteremia/septicemia, central nervous system infections, ear, nose, and throat infections and gastrointestinal infections. Cervical cerclage in our institution is typically performed using the transvaginal McDonald technique, which represents the standard approach in most cases. Cerclage placement is generally performed during the early second trimester in women with a history of cervical insufficiency or following ultrasound identification of cervical shortening. Due to the retrospective nature of the study and the long study period, detailed information regarding the exact indication for cerclage and timing of placement was not consistently available in the database.

Data were collected from the computerized hospitalization database of SUMC (“Demog-ICD9”), the computerized perinatal database of the obstetrics and gynecology department. Experienced medical secretaries routinely review the information before entering it into the database. Coding is performed after assessing medical and perinatal records as well as routine hospital documents.

Statistical analysis was performed using SPSS (version 24) and STATA (version 12.0) software. Differences between the groups were assessed using the χ^2^ test, *t* test, in accordance with the variable type and its distribution. The primary Kaplan–Meier survival curves were used to compare cumulative hospitalization incidences over time, and the differences were analyzed using the log-rank test. To establish an association between infectious diseases and future cumulative hospitalization incidence, while controlling for potential confounders, we used a secondary multivariate Cox proportional hazards model. Covariates included in the multivariable model were selected based on clinical relevance and prior literature, including gestational age, obesity and diabetes. This model included variables which may act as mediators, and was, therefore, considered exploratory. The proportional hazards assumption was evaluated and confirmed prior to model interpretation. *P*-values < 0.05 were considered statistically significant.

**Table 1:** Maternal and obstetrical outcomes, according to pregnancies with or without cervical cerclage

Maternal Characteristic/Pregnancy Outcome	No Cerclage *n* = 356,356	Cerclage *n* = 1416	OR	*P* value
Cesarean delivery (%)	13.9	29.0	2.5 (2.2–2.8)	< 0.001
Low birth weight (%)	6.9	18.7	3.1 (2.7–3.5)	< 0.001
GDM (%)	4.7	11.4	2.6 (2.2–3.0)	< 0.001
Hypertensive disease (%)	4.7	6.0	1.3 (1.0–1.6)	0.02
Perinatal mortality (%)	0.8	2.3	3.0 (2.1–4.2)	< 0.001
SGA (%)	4.6	3.3	0.7 (0.5–0.9)	0.02
Preterm delivery 34–37 weeks’ gestation (%)	6.8	24.9	4.5 (4.0–5.1)	< 0.001
Preterm delivery 28–34 weeks’ gestation (%)	1.6	8.6	5.8 (4.8–7.0)	< 0.001
Placental Abruption (%)	0.5	0.9	1.7 (1.0–3.0)	0.032

## Results

During the study period, 356,356 offspring met the inclusion criteria. Constituted in the analysis, 1,416 of them (0.4%) were following pregnancies with cervical cerclage.

These pregnancies exhibited distinct characteristics (Table 1), including higher rates of gestational diabetes (11.4% vs. 4.7%, *p* < 0.001), higher rates of preterm delivery in 34–37 gestational weeks (24.9% vs. 6.8%, *p* < 0.001) and in 28–34 gestational weeks (8.6% vs 1.6%, *p* < 0.001), a higher likelihood of cesarean deliveries (29.0% vs. 13.9%, *p* < 0.001), and perinatal mortality (2.3% vs 0.8%, *p* < 0.001).

**Fig. 1:**
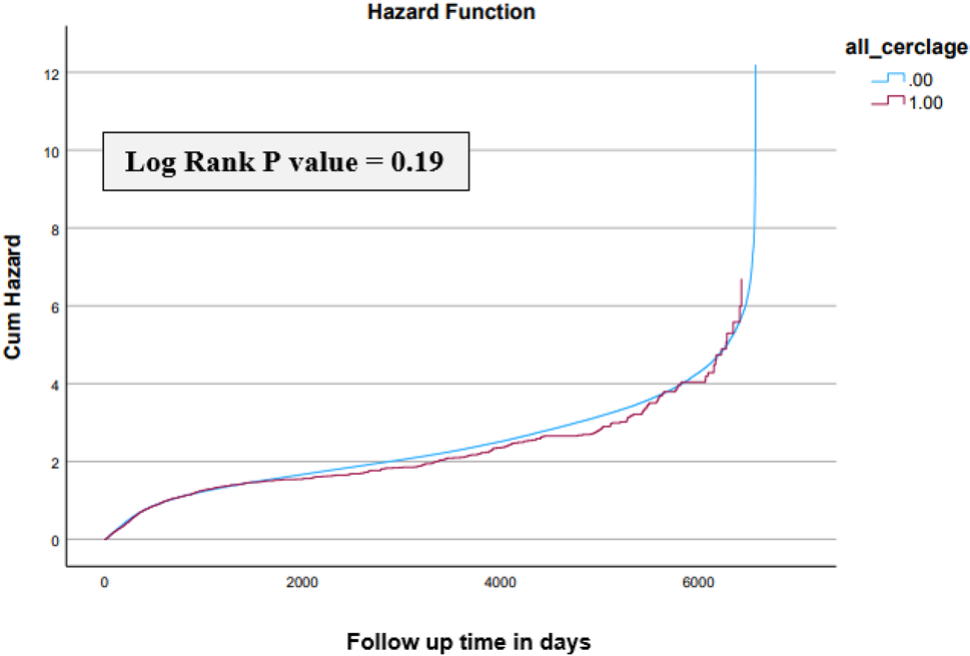
Cumulative incidence of infectious morbidity in offspring according to presence of cervical cerclage during pregnancy or not

The total infectious morbidity was comparable between the two study groups (OR 1.0, 95% CI 0.9–1.1; *p* = 0.369, Table 2). The primary Kaplan–Meier survival analysis demonstrated no significant difference in cumulative infectious morbidity between groups (log-rank test *p*-value = 0.19, Fig. 1). In a secondary multivariable Cox model, adjusting for confounders including gestational age, obesity and diabetes, cerclage exposure was associated with a modest reduction in infectious morbidity (adjusted HR 0.9, 95% CI 0.87–0.99, *p* = 0.036).

**Table 2:** Offspring long-term infectious morbidity after pregnancy with and without cervical cerclage

Infectious Morbidity	No Cerclage *n* = 356,356	Cerclage *n* = 1416	OR (95% CI)	*P* value
Respiratory infections (%)	176,546 (49.7)	730 (51.6)	1.1 (0.09–1.2)	0.173
Viral infections (%)	11,450 (3.2)	42 (3.0)	0.9 (0.7–1.2)	0.581
Ophthalmic infections (%)	6197 (1.7)	25 (1.8)	1.0 (0.7–1.5)	0.955
Skin infections (%)	15,672 (4.4)	63 (4.4)	1.0 (0.8–1.3)	0.951
Bacterial infections (%)	5171 (1.4)	20 (1.4)	0.9 (0.7–1.2)	0.889
Neonatal infections (%)	4637 (1.3)	21 (1.5)	1.1 (0.7–1.7)	0.559
Bacteremia/Septicemia (%)	3181 (0.9)	11 (0.8)	0.9 (0.5–1.6)	0.634
Central nervous system infections (%)	1849 (0.5)	8 (0.6)	1.0 (0.5–2.1)	0.818
Ear, nose and throat infections (%)	23,028 (6.5)	110 (7.8)	1.2 (0.9–1.4)	0.051
Gastrointestinal infections (%)	10,494 (3.0)	48 (3.4)	1.1 (0.9–1.5)	0.337
Total infectious morbidity (%)	195,557 (55.1)	797 (56.3)	1.0 (0.9–1.1)	0.369

## Discussion

This large population-based cohort study evaluated the long-term infectious morbidity in offspring born after pregnancies with cervical cerclage compared to those without. The primary finding of the study is that cerclage was not associated with an increased risk of infectious morbidity during childhood. While unadjusted analyses demonstrated similar infection rates between groups, a modest association with reduced infectious morbidity was observed after adjustment for selected confounders, such as gestational age, obesity, and diabetes. A secondary analysis identified distinct obstetrical characteristics in the cerclage group, including higher rates of gestational diabetes, cesarean delivery, and preterm birth.

The clinical interpretation of this finding requires careful consideration. The effect size observed in the adjusted analysis was small, and the confidence interval was close to unity. Given the large sample size of the cohort, statistically significant findings may occur even when differences are clinically modest. Therefore, these results should not be interpreted as evidence that cerclage confers a definitive protective effect against infections in offspring.

One possible explanation for the observed association relates to gestational age. Previous research primarily emphasized the gestational age extension afforded by cerclage as the primary mechanism for improved neonatal outcomes. It is well established that each additional week of gestation contributes to maturation of the fetal immune system, particularly adaptive immunity, which reduces susceptibility to infections such as respiratory and gastrointestinal illnesses during infancy and early childhood [16–20]. These findings are consistent with our data. However, in our adjusted analysis—where gestational age was accounted for—the protective effect persisted. This indicates that cerclage may exert effects beyond mechanical prolongation of pregnancy. However, residual confounding related to prematurity cannot be entirely excluded.

Another theoretical explanation relates to maternal immune responses during pregnancy. The presence of a cervical suture represents a foreign body that may induce localized inflammatory responses in the cervix [21, 22]. While historically viewed as a potential risk factor for infection, this low-grade, controlled inflammation may paradoxically enhance fetal immune development by promoting hematopoietic stem cell emergence and priming innate immunity [23–26]

Experimental studies have demonstrated that maternal immune activation during pregnancy can influence fetal immune development and postnatal immune responses [7–11]. However, this hypothesis remains speculative in the context of cervical cerclage and was not directly evaluated in the present study.

Another explanation should also be considered. Cervical shortening and the clinical indications for cerclage may be associated with underlying subclinical infection or inflammation [1–5]. Therefore, immune activation during pregnancy could reflect the maternal response to these conditions rather than a direct effect of the cerclage as a foreign body. Because our dataset did not include information on maternal infection status at the time of cerclage placement, we were unable to distinguish between these mechanisms, and the observed association should be interpreted cautiously.

In contrast, some studies have raised concerns about cerclage and its potential to disrupt the vaginal microbiota, thereby increasing the risk of ascending infection [26, 27]. For instance, research by Xiao et al. (2023) and Vargas et al. (2022) demonstrated that cerclage placement can reduce the dominance of protective Lactobacillus species and increase colonization by pathogenic bacteria. These changes are associated with adverse pregnancy outcomes and may theoretically pose a risk to neonatal health [28, 29]. However, these studies primarily focused on short-term pregnancy outcomes rather than long-term childhood health.

This study has several important strengths. The large population-based cohort and extended follow-up period allowed evaluation of long-term outcomes with substantial statistical power. In addition, the linkage of hospital and community medical databases enabled comprehensive capture of infectious diagnoses during childhood. The unique regional healthcare structure, in which most patients receive care within a single integrated healthcare system, also reduces loss to follow-up.

Nevertheless, several limitations should be acknowledged. The retrospective design introduces the possibility of misclassification or coding errors. Furthermore, important clinical variables such as the indication for cerclage, maternal infection status at the time of placement, antibiotic exposure, maternal smoking, body mass index, and socioeconomic factors were not available in the dataset. These variables may influence infection risk and could contribute to residual confounding. Confounding by indication represents an additional important limitation. Pregnancies requiring cerclage represent a selected high-risk population characterized by higher rates of preterm birth, cesarean delivery, gestational diabetes, hypertensive disorders, and perinatal mortality. Although our statistical models adjusted for selected confounders, unmeasured clinical factors may still influence the association observed in this study. Furthermore, some infections recorded in outpatient databases may reflect healthcare-seeking behavior. An important methodological consideration relates to adjustment for variables on the causal pathway. Gestational age may function as a mediator in the relationship between cerclage and long-term infectious morbidity, as cerclage is intended to prolong gestation. Adjustment for such variables may introduce over adjustment bias and potentially generate spurious associations. Accordingly, the primary analysis in this study was based on unadjusted time-to-event comparisons, which demonstrated no significant association. The modest association observed only after multivariable adjustment, including mediating variables, should, therefore, be interpreted with caution and may reflect model specification rather than a true causal effect. Another limitation relates to the definition of infectious morbidity. The outcome included a broad spectrum of diagnoses recorded in hospital and outpatient settings. While this approach allowed comprehensive capture of infectious events, some outpatient diagnoses may reflect healthcare utilization patterns rather than clinically severe disease. Additionally, odds ratios were used to describe differences in several outcomes with relatively high prevalence. In such situations, odds ratios may overestimate the magnitude of associations compared with risk ratios, and, therefore, the effect sizes should be interpreted cautiously. In addition, detailed microbiological data were not available in the database. Information regarding vaginal microbiome composition, including the presence of bacterial vaginosis or specific organisms such as Gardnerella or Ureaplasma, as well as the relative abundance of protective Lactobacillus species, was not recorded. Such data could help clarify whether immune activation observed in cerclage pregnancies is related to the foreign body itself or to underlying infection. Finally, the relatively low prevalence of cervical cerclage exposure in the cohort (0.4%) may introduce potential model instability and residual confounding despite the large overall sample size.

## Conclusion

In this large population-based cohort, cervical cerclage was not associated with increased long-term infectious morbidity in offspring. A modest association with reduced infectious morbidity was observed after adjustment for selected confounder. However, this finding should be interpreted cautiously given the observational design and potential residual confounding. Further studies are needed to better understand the mechanisms underlying this association and to evaluate long-term outcomes in children exposed to cerclage during pregnancy.

## Data Availability

No datasets were generated or analysed during the current study.

## References

[1] Goldenberg RL, Culhane JF, Iams JD, Romero R (2008) Epidemiology and causes of preterm birth. Lancet 371(9606):75–84 18177778 10.1016/S0140-6736(08)60074-4 PMC7134569

[2] Berghella V, Rafael TJ, Szychowski JM, Rust OA, Owen J (2011) Cerclage for short cervix on ultrasonography in women with singleton gestations and previous preterm birth: a meta-analysis. Obstet Gynecol 117(3):663–671 21446209 10.1097/AOG.0b013e31820ca847

[3] Alfirevic Z, Stampalija T, Roberts D, Jorgensen AL (2012) Cervical stitch (cerclage) for preventing preterm birth in singleton pregnancy. Cochrane Database Syst Rev 4:CD008991 10.1002/14651858.CD008991.pub2 22513970

[4] American College of Obstetricians and Gynecologists (2014) Practice Bulletin No. 142: cerclage for the management of cervical insufficiency. Obstet Gynecol 123(2):372–379. 10.1097/01.AOG.0000443276.68274.cc 24451674 10.1097/01.AOG.0000443276.68274.cc

[5] Shennan AH, Story L (2022) Royal College of Obstetricians, Gynaecologists. Cervical Cerclage: Green-top Guideline No. 75. BJOG 129(7):1178–1210. 10.1111/1471-0528.17003 35199905 10.1111/1471-0528.17003

[6] Rafael TJ, Berghella V, Alfirevic Z (2014) Cervical stitch (cerclage) for preventing preterm birth in multiple pregnancy. Cochrane Database Syst Rev 10:009166 10.1002/14651858.CD009166.pub2 PMC10629495 25208049

[7] Moawad GN, Tyan P, Bracke T et al (2018) Systematic review of transabdominal cerclage placed via laparoscopy for the prevention of preterm birth. J Minim Invasive Gynecol 25(2):277–2868 28797657 10.1016/j.jmig.2017.07.021

[8] Debbs RH, DeLa Vega GA, Pearson S et al (2007) Transabdominal cerclage after comprehensive evaluation of women with previous unsuccessful transvaginal cerclage. Am J Obstet Gynecol 197(3):317.e1-317.e4 17826436 10.1016/j.ajog.2007.06.060

[9] Lim AI et al (2021) Prenatal maternal infection promotes tissue-specific immunity and inflammation in offspring. Science 373(6555):eabf3002 34446580 10.1126/science.abf3002

[10] Gleditsch DD, Shornick LP, Van Steenwinckel J et al (2014) Maternal inflammation modulates infant immune response patterns to viral lung challenge in a murine model. Pediatr Res 76(1):33–40 24727945 10.1038/pr.2014.57

[11] Mukherjee S, Allen RM, Lukacs NW et al (2012) STAT3-mediated IL-17 production by postseptic T cells exacerbates viral immunopathology of the lung. Shock 38(5):515–523 23042197 10.1097/SHK.0b013e31826f862c PMC3475732

[12] Witkin SS (2015) The vaginal microbiome, vaginal anti-microbial defence mechanisms, and the clinical challenge of reducing infection-related preterm birth. BJOG 122(2):213–218 25316066 10.1111/1471-0528.13115

[13] Lacroix G, Gouyer V, Gottrand F, Desseyn JL (2020) The cervicovaginal mucus barrier. Int J Mol Sci 21(21):8266 33158227 10.3390/ijms21218266 PMC7663572

[14] Leitich H, Kiss H (2007) Asymptomatic bacterial vaginosis and intermediate flora as risk factors for adverse pregnancy outcome. Best Pract Res Clin Obstet Gynaecol 21(3):375–390 17241817 10.1016/j.bpobgyn.2006.12.005

[15] Kindinger LM et al (2016) Relationship between vaginal microbial dysbiosis, inflammation, and pregnancy outcomes in cervical cerclage. Sci Transl Med 8(350):350102 10.1126/scitranslmed.aag1026 27488896

[16] Melville JM, Moss TJM (2013) The immune consequences of preterm birth. Front Mol Neurosci 7:79 10.3389/fnins.2013.00079 PMC3659282 23734091

[17] Miller JE, Hammond GC, Strunk T et al (2016) Association of gestational age and growth measures at birth with infection-related admissions to hospital throughout childhood: a population-based, data-linkage study from Western Australia. Lancet Infect Dis 16(8):952–961 27052469 10.1016/S1473-3099(16)00150-X

[18] Davidesko S, Wainstock T, Sheiner E, Pariente G (2020) Long-term infectious morbidity of premature infants: is there a critical threshold? J Clin Med 9(9):3008 32961963 10.3390/jcm9093008 PMC7563528

[19] Padeh E, Wainstock T, Sheiner E et al (2019) Gestational age and the long-term impact on children’s infectious urinary morbidity. Arch Gynecol Obstet 299(2):385–392 30515555 10.1007/s00404-018-4973-4

[20] Imterat M, Wainstock T, Moran-Gilad J et al (2019) The association between gestational age and otitis media during childhood: a population-based cohort analysis. J Dev Orig Health Dis 10(2):214–220 30223907 10.1017/S2040174418000685

[21] Cai S, Wu Y, Zeng L, Ding Y (2022) Effects of vaginal microecology and immunity on the pregnancy outcome of cervical cerclage. BMC Womens Health 22(1):167 35568847 10.1186/s12905-022-01751-9 PMC9107276

[22] Fang J, Lin Y, Chen Z et al (2023) The association of inflammatory markers with maternal-neonatal outcome after cervical cerclage. J Inflamm Res 16:245–255 36698755 10.2147/JIR.S393666 PMC9869902

[23] Apostol AC, López DA, Lebish EJ, et al. Prenatal inflammation perturbs fetal hematopoietic development and causes persistent changes to postnatal immunity. bioRxiv. Published May 8, 2022. 10.1101/2022.05.08.491095 10.1016/j.celrep.2022.111677 PMC10184520 36417858

[24] Espin-Palazon R, Weijts B, Mulero V, Traver D (2018) Proinflammatory signals as fuel for the fire of hematopoietic stem cell emergence. Trends Cell Biol 28(1):58–66 28882414 10.1016/j.tcb.2017.08.003

[25] Mariani SA, Li Z, Rice S et al (2019) Pro-inflammatory aorta-associated macrophages are involved in embryonic development of hematopoietic stem cells. Immunity 50(6):1439-1452.e5 31178352 10.1016/j.immuni.2019.05.003 PMC6591003

[26] Vidal MS Jr, Menon R (2023) In utero priming of fetal immune activation: myths and mechanisms. J Reprod Immunol 157:103922 36913842 10.1016/j.jri.2023.103922 PMC10205680

[27] Quinn M (1993) Final report of the MRC/RCOG randomised controlled trial of cervical cerclage. Br J Obstet Gynaecol 100(12):1154–1155 8297859 10.1111/j.1471-0528.1993.tb15198.x

[28] Xiao Y, Huang S, Yu W et al (2023) Effects of emergency/nonemergency cervical cerclage on the vaginal microbiome of pregnant women with cervical incompetence. Front Cell Infect Microbiol 13:1072960 36968117 10.3389/fcimb.2023.1072960 PMC10034410

[29] Vargas M, Yañez F, Elias A et al (2022) Cervical pessary and cerclage placement for preterm birth prevention and cervicovaginal microbiome changes. Acta Obstet Gynecol Scand 101(12):1403–1413 36168933 10.1111/aogs.14460 PMC9812209

